# Complementary and alternative medicines chosen for specific health problems: Internet survey using the I-CAM-Q in Japan: A STROBE-compliant cross-sectional study

**DOI:** 10.1097/MD.0000000000031098

**Published:** 2022-10-14

**Authors:** Ryo Tabata, Harutaka Yamaguchi, Yoshihiro Ookura, Kenji Tani

**Affiliations:** a Department of General Medicine, University of Tokushima Graduate School of Biomedical Sciences, Tokushima, Japan; b Department of General Medicine and Primary Care, Tokushima University Hospital, Tokushima, Japan.

**Keywords:** complementary and alternative medicine, health problems, I-CAM-Q, Japan

## Abstract

This study investigated the different complementary and alternative medicines (CAMs) adopted by people in Japan, as well as the health problems treated with CAMs. Understanding more about this topic will facilitate the appropriate incorporation of CAMs into conventional medicine when treating health problems. Data were collected through an online survey based on the International Questionnaire to Measure Use of Complementary and Alternative Medicine (I-CAM-Q). The study examined CAM utilization among people aged 20 years or older; 164 valid responses were collected (18.9%). We adopted a cross-sectional design. We then compared the relationships between the specific health problems reported by participants, their self-help practices, and the kinds of healthcare specialists consulted. We also examined participants’ reasons for using CAMs and their responses regarding the usefulness of the CAMs adopted. We found that self-help/CAM practices differed for specific health problems. Participants with musculoskeletal and heart problems were more likely to use poultices. Those with respiratory and digestive problems were more likely to practice yoga, tai chi, and qigong. Those with digestive and neurological problems were more likely to use aromatherapy. The I-CAM-Q questionnaire also revealed the purpose and usefulness of the CAMs utilized as well as the participants’ attitudes regarding conventional medicine. The study showed that participants find physicians less helpful than other options for treating their health problems. Additionally, when asked whether it was helpful to consult with a specific professional for health problems, there was a higher percentage of participants who answered “Very helpful” for professionals, like massage, judo, acupuncture, and moxibustion therapists, than for physicians. The results of this study will help to inform medical providers of the most appropriate types of CAMs for dealing with various health problems.

## 1. Introduction

The definition of complementary and alternative medicine (CAM) by the Cochrane Complementary Medicine Field covers a broad domain of healing resources that encompasses all health systems, modalities, and practices, as well as their accompanying theories and beliefs. Furthermore, CAM is suggested as an alternative to those health systems intrinsic to the politically dominant health system of a given society or culture in a specific historical period.^[1]^ While it is true that conventional medicine plays a major role in the diagnosis and treatment of many diseases, many disciplines have failed to achieve their full potential. Complementary medicines involve therapies that are used in combination with conventional medicines, while alternative medicines are those used in place of conventional medicines. One report revealed that 69% of their respondents said that there should be more collaboration between conventional and complementary medicines.^[2]^ Furthermore, CAMs are now being used worldwide. The percentage of people who have used CAM therapies at some point in their lives was found to be 26.3% in the United Kingdom,^[3]^ 34% in Norway, 45% in Denmark,^[4]^ 38.3% in the United States (US),^[5]^ 74.2% in Korea,^[6]^ and 76% in Japan.^[7]^

Although CAMs are used internationally, with various studies being conducted on their efficacy, it is not easy to compare the extant studies on these therapies because of the diversity of research methods and the various definitions of CAMs used across different countries and regions.^[8–10]^ Furthermore, we found that studies on CAMs are influenced by researchers’ unique survey methodology and questionnaires, even when conducted in the same country and region. As a result, a standardized questionnaire named the International Questionnaire to Measure the Use of Complementary and Alternative Medicine (I-CAM-Q) was created at an international workshop on CAMs, which was held in Norway in 2006.^[11]^ I-CAM-Q is designed to reduce questionnaire bias and provide accurate international comparisons of CAM usage among populations. Several I-CAM-Q studies have already been published,^[2,10,12–31]^ with the possibility of more international comparative research to be conducted in the future.

In Japan, there have been 4 published reports of surveys that used the I-CAM-Q. One focused on the general population of the country,^[32]^ while the other 3 specifically involved patients within medical institutions.^[22,28,29]^ However, there are currently no reports using the I-CAM-Q that investigate the relationship between peoples’ health problems and their specific self-help practices in Japan. The I-CAM-Q is an effective way to survey Japanese participants regarding this topic because it is internationally standardized. Furthermore, I-CAM-Q not only indicates which self-help CAM practice is being used for each health problem but also clarifies the reason and purpose behind the therapy chosen.

## 2. Methods

### 2.1. Study design and population

The survey was administered, from June 11 to July 1, 2020, to members of the Japan Association of Medical Body Contouring (JAMBC), who were aged 20 years or older. As of July 1, 2020, the total number of JAMBC members was 1056. The founding principle of the JAMBC is “halving the number of lifestyle-related diseases and contributing to people’s health and prosperity in Japan.” Furthermore, this association conducts academic research on dieting, constitutional improvements, dietary improvements, and preventive medicine.

### 2.2. Questionnaire development and data collection

This survey was conducted online, using Google Limited Liability Company’s “Google Forms” program. The I-CAM-Q is designed to reduce questionnaire bias and provide accurate international comparisons of peoples’ use of CAMs. The I-CAM-Q was prepared in Japanese, based on how it was used in a previous survey conducted in Japan.^[3]^ The questions were then adjusted to the Google Forms format. The link to the Google Form was emailed to the participants, with the consent form attached. The questions covered the following domains: sex, age, health status, and health problems. For sex, “Male,” “Female,” and “Other” were the available options, with participants selecting one. Age was entered freely. With regard to participants’ health status, 1 option could be selected from the responses provided, namely, “Very good,” “Good,” “Not bad,” and “Bad.” In terms of participants’ health problems, participants could select multiple responses, including “Musculoskeletal,” “Heart,” “Respiratory,” “Neurological,” “Digestive,” “Gynecological,” “Urinary tract,” “Kidney,” “Endocrine,” “Skin,” “Cancer,” “Allergy,” “Chronic pain,” “Mental,” “Ear,” “Dental,” and “Others.” The “Others” option was left open-ended to elicit more detailed answers.

Participants were also asked which kinds of specialists they had consulted with for their health problems within the past year, the number of consultations within the previous 3 months, the reason for their most recent consultation (e.g., acute illness, long-term illness, improving their well-being, others), and whether the consultation was useful (e.g., very helpful, somewhat, not at all, uncertain). The specialists were selected from a series of multiple choices: “Physicians,” “Nurses,” “Judo therapists,” “Rehabilitation therapists,” “Massage therapists,” “Acupuncture and Moxibustion therapists,” “Spiritual healers,” “Fortune tellers,” and “Others.” The “Others” option was open-ended for more detailed answers.

When a participant had consulted a physician within the preceding year, they were asked what CAM was advised and utilized, the number of times they had consulted with the physician in the previous 3 months, the most recent reason for the advice or treatment (e.g., acute illness, long-term illness, improving their well-being, or other reasons), and whether it was useful (very helpful, somewhat, not at all, uncertain). The CAMs advised or used as a treatment by the physicians were selected from a series of multiple choices, including the following: “Herbal medicine,” “Supplements,” “Acupuncture and Moxibustion,” “Exercise therapy,” “Massages,” “Hot-spring cure,” “Others.” The “Others” option was again open-ended for more detailed answers.

They were then asked what CAM they had chosen for themselves within the previous year, the number of times they had used it within the preceding 3 months, the most recent reason for it (same options as before), and whether it was useful (same options as before). The CAMs were selected from multiple choices, including “Dietary therapy,” “Exercise therapy,” “Hot-spring cure,” “Using poultices,” “Zen and Meditation,” “Yoga, Tai Chi, and Qigong,” “Praying for recovery from illness and health,” “Aromatherapy,” and “Others.” Similar, to before, the “Others” option was left open-ended for more detailed answers. “Others” also had 2 items, —“Other 1” and “Other 2”—with the result of adding the 2 items then being shown. It must be noted that whether prayers should be included as a CAM is a matter of debate. Currently, there is no universally accepted definition of prayer as a CAM. However, Eardley et al^[33]^ stated that, even if linking spiritual healing to religion was not the original intent of the survey, a participant’s own interpretation of spiritual healing should always be considered valid. Prayer was included in the current questionnaire, as it has been used as an example of CAM in several previous studies.^[12,19,34]^

Information regarding the herbal medicines, herbs, or supplements that the participants had taken in the past year, whether they were still using them, the most recent reason for using them (same options as before), and their usefulness (same options as before) were also collected. The names of the products were then entered freely by the participants.

### 2.3. Reasons and usefulness

Respondents were asked to select 1 reason for seeking out CAMs from a list of available responses, including “Acute illness,” “Long-term illness,” “Improving their well-being,” and “Others.” “Acute illness” was defined as an acute illness or disorder that was cured within 1 month. “Long-term illness” was defined as a chronic illness or disorder that had not been cured within 1 month. “Improving their well-being” was defined as the maintenance of one’s health over time. From each of the available responses outlining the CAMs’ usefulness, including “Very helpful,” “Somewhat,” “Not at all,” and “Uncertain,” the participants were instructed to select one.

### 2.4. Statistical analyses

All statistical analyses were performed using IBM SPSS Statistics version 24. A comparison between the participant groups was made using a Chi-squared test for categorical variables as well as a Mann–Whitney *U* test for ordinal variables. The data were presented either in numbers as percentages or as odds ratios (ORs) with 95% confidence intervals (Cis). The results were considered significant when the *P* value was <.05.

The study protocol was approved by the Tokushima University Hospital Research Ethics Board (No. 3705). All participants read the consent information document and provided their consent to participate in this study.

## 3. Results

We sent an email asking for participants’ health status to all the 991 registered JAMBC members (215 males and 776 females), with 869 members (95.4%) receiving this communication and 168 members (19.3%) responding. After excluding responses with any unanswered questions, 164 (18.9%) members were included in the final analysis. The demographics, health statuses, and CAM usage rates of the participants are shown in Table 1. Of the participants, 36 (22.0%) were males, and 128 (78.0%) were females. Most participants, 64 to be precise (39.0%), were in their 50s, followed by 54 (32.9%) in their 40s, 24 (14.6%) in their 30s, 13 (7.9%) in their 60s, 7 (4.3%) in their 20s, and finally, 2 (1.2%) in their 70s. In terms of participants’ health statuses, 43 (26.2%), 94 (57.3%), 24 (14.6%), and 3 (1.8%) responded with “Very good,” “Good,” “Not bad,” and “Bad,” respectively. The number of participants who answered that they had used CAM in this survey was 157, making our sample’s usage rate 95.7%. We then divided the participants into 2 groups: those who responded as having one or more health problems (i.e., those with health problems) and those who did not have any health problems (i.e., those with no health problems). In terms of the sex differences, 22 (61.1%) male and 99 (77.3%) female participants reportedly had health problems, with the latter tending to report having more health problems overall, although this finding was not significant (*P* = .05). According to age group, 3 participants in their 20s (42.9%), 21 (87.5%) in their 30s, 43 (79.6%) in their 40s, 44 (68.8%) in their 50s, 10 (76.9%) in their 60s, and none in the 70s had any health problems. There was no significant difference found between the age groups herein (*P* = .214). All participants who responded as having no health problems also reported having a positive health status (either “Very good” or “Good”). There was a significant difference found in the health statuses between those with health problems and those without any (*P* < .001). In addition, we found no significant difference between participants’ CAM usage and their reported health problems (*P* = .306).

**Table 1:** Demographics, health statuses, and CAM usage rate.

		N = 164	Health problems		*P* value**
			Yes (N = 121)	No (N = 43)	
Sex	Male	36 (22.0)	22 (61.1)	14 (38.9)	.050
	Female	128 (78.0)	99 (77.3)	29 (22.7)	
Age (yr)	20~29	7 (4.3)	3 (42.9)	4 (57.1)	.214
	30~39	24 (14.6)	21 (87.5)	3 (12.5)	
	40~49	54 (32.9)	43 (79.6)	11 (20.4)	
	50~59	64 (39.0)	44 (68.8)	20 (31.3)	
	60~69	13 (7.9)	10 (76.9)	3 (23.1)	
	70~79	2 (1.2)	0 (0.0)	2 (100)	
Health status	Very good	43 (26.2)	22 (51.2)	21 (48.8)	<.001*
	Good	94 (57.3)	72 (76.6)	22 (23.4)	
	Not bad	24 (14.6)	24 (100)	0 (0.0)	
	Bad	3 (1.8)	3 (100)	0 (0.0)	
CAM†	Use	157 (95.7)	117 (74.5)	40 (25.5)	.306
	No use	7 (4.3)	4 (57.1)	3 (42.9)	

Values are presented as number (%).

*Significance at *P* < .05.

** *P* values for Sex and CAM were calculated using chi-square test; *P* values for participants’ age and health status were calculated using the Mann–Whitney *U* test.

† CAM = complementary and alternative medicines.

**Figure 1.**
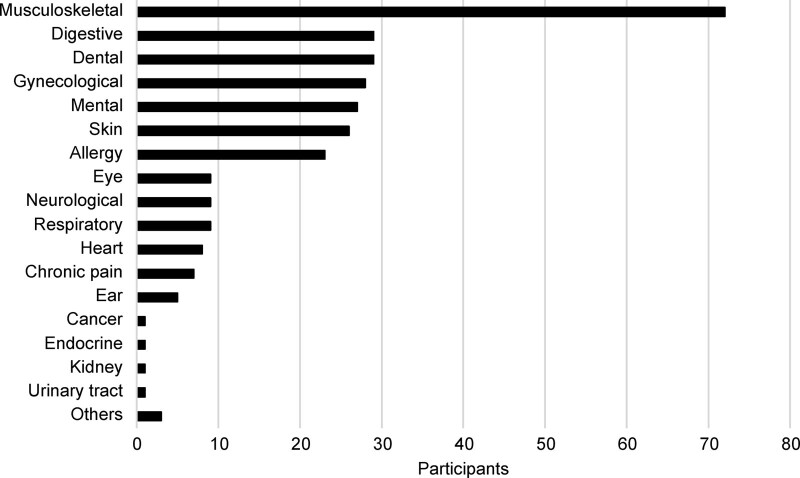
Health problems.

The participants’ health problems are shown in Figure 1. The most common health problems were musculoskeletal problems reported by 72 (43.9%) participants, followed by digestive problems by 29 (17.7%) participants, tooth problems by 29 (17.7%) participants, gynecological problems by 28 (17.1%) participants, mental health problems by 27 (16.5%) participants, skin problems by 26 (15.9%) participants, and allergies by 23 (14.0%) participants. A total of 49 (29.9%) respondents had 1 health problem, 31 (18.9%) had two, 16 (9.8%) had 3, and 25 (15.2%) had 4 or more.

The types of specialists that participants had consulted with for their respective health problems are shown in Table 2. The majority of participants, 71 (43.3%), had consulted with a physician for their health problems, followed by 29 (17.7%) who had consulted with massage therapists, 20 (12.2%) who had consulted with acupuncture and moxibustion therapists, 18 (11.0%) who had consulted with judo therapists, and those who had consulted with nurses coming to 9 (5.5%). In the case of acute illnesses, consulting with a physician (28.2%) was the most common response, followed by consulting with a massage therapist (24.1%). Among participants who underwent consultations for a long-term illness, the highest percentage included those who had consulted with a judo therapist at 50.0%, with those who had consulted with acupuncture and moxibustion therapists or rehabilitation therapists being the next highest, at 40%. In terms of improving their well-being, the highest number of participants consulted with nurses (88.9%), followed by those who had consulted with rehabilitation therapists (40.0%) and then massage therapists (37.9%). When asked if they had found the consultation to be useful, the majority of the participants who responded with “Very helpful” (81.8%) had consulted with spiritual healers. Acupuncture and moxibustion therapists, rehabilitation therapists, and judo therapists accounted for 65.0%, 60.0%, and 52.6% of responses herein, respectively. The lowest percentage for this response was 25.4%, which included those who had consulted physicians. A total of 50 respondents (30.5%) had consulted with one type of specialist, 31 (18.9%) had consulted with 2 types, 15 (9.1%) had consulted with 3 types, and, finally, 10 (6.1%) had consulted with 4 or more types of specialists.

**Table 2:** Classification and usefulness of specialists consulted for health problems.

Specialists	N = 164	Reason for consulting the specialist					Usefulness of consultation			
		Acute illness	Long-term illness		Improve well-being	Others	Very helpful	Somewhat helpful	Not at all	Uncertain
Physicians	71 (43.3)	20 (28.2)	25 (35.2)	13 (18.3)		13 (18.3)	18 (25.4)	38 (53.5)	6 (8.5)	9 (12.7)
Massage therapists	29 (17.7)	7 (24.1)	9 (31.0)	11 (37.9)		2 (6.9)	12 (41.4)	13 (44.8)	2 (6.9)	2 (6.9)
Acupuncture and moxibustion therapists	20 (12.2)	4 (20.0)	8 (40.0)	7 (35.0)		1 (5.0)	13 (65.0)	7 (35.0)	0 (0.0)	0 (0.0)
Judo therapists	18 (11.0)	4 (20.0)	10 (50.0)	4 (20.0)		2 (10.0)	10 (52.6)	9 (47.4)	0 (0.0)	0 (0.0)
Spiritual healers	12 (6.7)	1 (8.3)	0 (0.0)	2 (16.7)		9 (75.0)	9 (81.8)	2 (18.2)	0 (0.0)	0 (0.0)
Fortunetellers	15 (9.1)	1 (7.1)	1 (7.1)	2 (14.3)		10 (71.4)	6 (42.9)	5 (35.7)	1 (7.1)	2 (14.3)
Nurses	9 (5.5)	1 (11.0)	0 (0.0)	8 (88.9)		0 (0.0)	4 (40.0)	3 (30.0)	3 (30.0)	0 (0.0)
Rehabilitation therapists	5 (3.0)	1 (20.0)	2 (40.0)	2 (40.0)		0 (0.0)	3 (60.0)	1 (20.0)	1 (20.0)	0 (0.0)
Others	18 (11.0)	1 (5.6)	2 (11.1)	9 (50.0)		6 (33.3)	11 (61.1)	7 (38.9)	0 (0.0)	0 (0.0)
None	58 (35.4)									

Values are presented as number (%).

The types of CAMs that respondents had received as treatment or were advised to use by physicians are shown below. Herbal medicine and exercise therapy had the highest number of responses, both at 8 (4.9%). None of the patients had received acupuncture or moxibustion. A total of 13 (7.9%) participants had received one type of CAM from their physician, 3 (1.8%) had received 2 types, 1 (0.6%) had received 3 types, and 1 (0.6%) had received 4 or more types.

The types of self-help practices utilized by respondents are shown in Table 3. The highest number of participants who had undergone diet therapy on their own was 105 (64.0%), followed by those who had undergone exercise therapy at 59 (36.0%), aromatherapy with 36 (22.0%) responses, and praying for recovery from an illness or improvement in one’s health coming to 33 (20.1%). The self-help practice used for treating an acute illness with the highest percentage of responses was the use of poultices with 62.5%. For long-term illnesses, too, the highest percentage of responses involved “Using poultices” (25.0%), followed by “Praying for recovery from an illness or improving one’s health” (18.2%), and “Exercise therapy” (15.3%). The majority of the participants (93.8%) practiced yoga, tai chi, and qigong to improve their overall well-being. In terms of self-help practices used to improve their well-being, 90.0% used a hot-spring cure, 82.9% used diet therapy, 78.0% used exercise therapy, 75.0% used aromatherapy, 72.2% used Zen and meditation, and 66.7% used prayer. When asked if their chosen self-help practices were useful, over 50% of the participants answered “Very helpful” practiced yoga, tai chi, qigong; dietary therapy; aromatherapy; and Zen and meditation. The lowest percentage of “Very helpful” responses comprised those surrounding the use of poultices (20.8%). Very few participants, 4 (1.2%), answered: “Not at all.” A total of 37 (22.6%) participants had undergone 1, 47 (28.7%) had undergone 2, 26 (15.9%) had undergone 3, and 27 (16.5%) had undergone 4 or more self-help practices involving CAMs.

**Table 3:** Type of self-help practices used and their usefulness.

Practice	N = 164	Reason for treatment				Usefulness of treatment			
		Acute illness	Long-term illness	Improve well-being	Others	Very helpful	Some what	Not at all	Uncertain
Dietary therapy	105 (64.0)	6 (5.7)	8 (7.6)	87 (82.9)	4 (3.8)	58 (55.2)	45 (42.9)	0 (0.0)	2 (1.9)
Exercise therapy	59 (36.0)	3 (5.1)	9 (15.3)	46 (78.0)	1 (1.7)	19 (32.2)	37 (62.7)	1 (1.7)	2 (3.4)
Aromatherapy	36 (22.0)	0 (0.0)	3 (8.3)	27 (75.0)	6 (16.7)	19 (52.8)	13 (36.1)	1 (2.8)	3 (8.3)
Praying for recovery from an illness and improvement of health	33 (20.1)	1 (3.0)	6 (18.2)	22 (66.7)	4 (12.1)	12 (36.4)	13 (39.4)	0 (0.0)	8 (24.2)
Poultices	24 (14.6)	15 (62.5)	6 (25.0)	2 (8.3)	1 (4.2)	5 (20.8)	19 (79.2)	0 (0.0)	0 (0.0)
Hot-spring cure	20 (24.0)	0 (0.0)	2 (10.0)	18 (90.0)	0 (0.0)	9 (45.0)	10 (50.0)	1 (5.0)	0 (0.0)
Zen and meditation	18 (11.0)	0 (0.0)	1 (5.6)	13 (72.2)	4 (22.2)	9 (50.0)	8 (44.4)	1 (5.6)	0 (0.0)
Yoga, Tai Chi, and Qigong	16 (9.8)	0 (0.0)	1 (6.3)	15 (93.8)	0 (0.0)	9 (56.3)	7 (43.8)	0 (0.0)	0 (0.0)
Others	26 (15.8)	1 (3.8)	1 (3.8)	23 (88.5)	1 (3.8)	19 (73.1)	4 (15.4)	0 (0.0)	3 (11.5)
None	27 (16.5)								

Values are presented as number (%).

We then compared the relationship between respondents’ specific health problems and their self-help practices, with the relevant ones being shown in Table 4. Those with musculoskeletal and heart problems were more likely to use poultices (Musculoskeletal OR: 4.78, 95% CI: 1.79-12.79, *P* = .001; Heart OR: 6.80, 95% CI: 1.57-29.38, *P* = .017). Those with respiratory and digestive problems were more likely to practice yoga, tai chi, and qigong (Respiratory OR: 5.46, 95% CI: 1.22-24.42, *P* = .045; Digestive OR: 4.46, 95% CI: 1.50-13.20, *P* = .010). Those with digestive and neurological problems were more likely to use aromatherapy (Digestive OR: 2.69, 95% CI: 1.13-6.40, *P* = .022; Neurological OR: 8.33, 95% CI: 1.97-35.25, *P* = .004). Furthermore, those with digestive problems were less likely to use the hot-springs cure (*P* = .016). Finally, those with skin problems were less likely to use exercise therapy (OR: 0.27, 95% CI: 0.09-0.84, *P* = .017).

**Table 4:** Relationship between health problems and self-help practices utilized.

Health problems		Self-help practices		OR*	95% CI†	*P* value‡
		Poultices				
		Yes	No			
Musculoskeletal	Yes	18**	54	4.78	1.79-12.79	.001
	No	6	86**			
Heart	Yes	4**	4	6.80	1.57-29.38	.017
	No	20	136**			
		Yoga, Tai Chi, Qigong				
		Yes	No			
Respiratory	Yes	3**	6	5.46	1.22-24.42	.045
	No	13	142**			
Digestive	Yes	7**	22	4.46	1.50-13.20	.010
	No	9	126**			
		Aromatherapy				
		Yes	No			
Digestive	Yes	11**	18	2.69	1.13-6.40	.022
	No	25	110**			
Neurological	Yes	6**	3	8.33	1.97-35.25	.004
	No	30	125**			
		Hot-spring cure				
		Yes	No			
Digestive	Yes	0	29**			.016
	No	20**	115			
		Exercise therapy				
		Yes	No			
Skin	Yes	4	22**	0.27	0.09-0.84	.017
	No	55**	83			

* OR: odds ratio.

† CI: confidence interval.

‡ *P* values were calculated using chi-square test.

** Significance at *P* < .05.

## 4. Discussion

This study was conducted on the use of CAMs in Japan using the I-CAM-Q, an international questionnaire, over the Internet. In particular, this research focused on the particulars of respondents’ CAM usage for the specific health problems that they were experiencing. The I-CAM-Q questionnaire also allowed us to examine both the respondents’ reasons for using as well as their perceived usefulness of each CAM. The following findings were observed: Participants with musculoskeletal and heart problems were more likely to use poultices. Furthermore, poultices were often used for acute and long-term illnesses. Participants with respiratory and digestive problems were more likely to practice yoga, tai chi, and qigong. Additionally, yoga, tai chi, and qigong were often used to improve participants’ well-being. Participants with digestive and nervous system problems were more likely to use aromatherapy. Similarly, aromatherapy was often used to improve the respondents’ overall well-being. Participants with digestive problems were less likely to use hot springs. Those with skin problems were less likely to undergo exercise therapy. Further, physicians were the most common specialists with whom respondents had consulted for their health problems. However, when asked about their usefulness, the percentage of those who answered “Very helpful” for physicians was the lowest. Following physicians, the most common health specialists consulted by the respondents were massage therapists, acupuncture and moxibustion therapists, and judo therapists. The percentage of those who answered “very helpful” for these categories was higher than that of physicians. Compared to previous studies, relatively fewer people consulted with nurses. In the first half of our investigation, we examined how effective the respondents felt the therapies were in treating each of their health problems. In the second half, we focused on the specialists with whom the participants had consulted.

Each therapy was found to be effective against each of the associated health problems. One of the first-line treatments suggested in the new guidelines for safe and effective pain management is poultices.^[35]^ This is a treatment that targets the location of the pain. Therefore, it is understandable that participants with musculoskeletal problems would use poultices. Furthermore, heart diseases are known to cause mood disorders in approximately 30% of patients.^[36]^ This is corroborated by the fact that, in Japan, cardiac exercise therapy has been reported to improve anxiety, depression, and patients’ quality of life (QOL) following acute myocardial infarction.^[37]^ In patients with heart failure, exercise therapy has also been reported to reduce their anxiety and depression and improve their overall QOL.^[38]^ In addition, exercise therapy may be necessary to manage pain. Poultices can also reduce the risk of systemic adverse events.^[35]^ As such, people with heart disease are more likely to be interested in exercise and to use more poultices.

Furthermore, yoga has been found to balance the autonomic nervous system by increasing parasympathetic activity and decreasing sympathetic activity.^[39]^ Multiple studies have shown that yoga has a profound effect on many psychological and physical factors, including peoples’ digestive symptoms, immune system function, blood pressure, mood, and anxiety, when compared to the use of psychotherapy or meditation alone.^[40]^ The practice of yoga also involves slow breathing techniques that improve one’s lung capacity and respiratory health.^[41]^ Thus, yoga might be more popular among participants with respiratory and digestive problems. As the nervous system influences both the endocrine and immune systems when performing mind-body (MB) exercises, MB-based interventions have the potential to improve one’s overall bodily functions and health. Tai chi, yoga, and qigong are often considered the most popular MB exercises.^[42]^

In general, aromatherapy is believed to help with symptoms of stress, chronic pain, nausea, and depression.^[43]^ In addition to the direct benefits of the oils involved, other positive outcomes have also been observed. For example, aromatherapy has been found to improve neurotransmission by inhibiting acetylcholinesterase and increasing acetylcholine in cholinergic neurons, meaning that the essential oils utilized can delay neurodegeneration and cognitive decline in illnesses like dementia.^[44]^ Due to these benefits, aromatherapy would be useful for participants with both digestive and nervous system problems.

Hot springs are often used by many people; hot spring treatment involves multiple people sitting in public baths. As such, people with difficulties in controlling defecation should refrain from using hot springs. Some participants might also be reluctant to go out to hot springs.

Some skin diseases may be aggravated by sunlight and sweat. Furthermore, some participants might be reluctant to go outside owing to cosmetic reasons and thus become less likely to use exercise therapy.

This study found that the perceived role of physicians in treating a variety of health problems is highly important. However, in a study among cancer patients in Japan, nearly 2-thirds of respondents had never consulted with their physicians about the use of CAMs. The main reason for this was that the physicians did not specifically mention using any CAM therapy.^[45]^ Several similar reports can also be cited here. For example, one large study conducted in the US in 1997 found that almost all patients interviewed had undergone CAM procedures previously. However, when the participants herein had consulted with their physicians regarding their symptoms, only one-third of them had discussed CAMs.^[43]^ For example, one study conducted in the US in 1997 found that participants interviewed had undergone CAM procedures previously. However, only one-third of the CAMs used by the participants discussed their physician.^[46]^ Another study by Richardson et al^[47]^ that investigated the reasons for the communication gap between physicians and patients regarding CAM therapies found that physicians feel that their patients would consider any discussion about CAM as unimportant. On the contrary, many patients believe that physicians do not understand the use of CAMs and would even refuse to use them. A lack of knowledge on CAMs among physicians is also a problem mentioned in the extant literature. One study in Turkey found that most general practitioners have received no education on CAMs.^[48]^ In a Japanese survey, most of the content surrounding CAMs that is taught in medical schools involves oriental medicine, including herbal medicine and acupuncture therapy.^[49]^ As such, there seems to be a bias in the education of CAM utilization. This could be the reason that the number of participants who responded that consulting with a physician was very helpful was the lowest among all the listed options. Based on our findings, we believe that physicians, who play a major role in the treatment of many health problems, should make an increased effort to gain sufficient knowledge about CAM usage while simultaneously communicating adequately with their patients about their CAM expectations.

In this study, health problems were also found to be frequently encountered by massage therapists. This is corroborated by the fact that massage is a popular CAM worldwide. For example, in a Swedish survey, one-third of the participants answered that they had previously consulted with a CAM provider, of which massage therapists were the most common.^[2]^ In another study, it was found that about one in ten participants in a rural area of Japan had consulted with massage therapists for various health problems.^[28]^ Generally speaking, a single massage session takes longer than a physician’s consultation. A Japanese study found that patients receiving CAM felt strongly that they were not given enough consultation time in regular physician practices.^[50]^ Patients, thus, prefer CAM by massage therapists because of the more in-depth human relationships formed. In this study, when asked whether it was helpful to consult with a specific professional for health problems, the percentage of participants who answered “Very helpful” was higher for professionals like massage therapists, judo therapists, and acupuncture and moxibustion therapists than for physicians. Listening to the patients and having heartfelt conversations with them during treatment have been thought to increase patient satisfaction herein.^[32]^ These results provide implications for strategies to improve the quality of general healthcare practices.

In this study, the number of participants who had consulted with nurses for their health problems was low. In one Korean study, about half of the participants had consulted with nurses about their health problems.^[21]^ In another study conducted in Japan, one-fifth of the participants had consulted with nurses for various health problems.^[32]^ In Japan, few people tend to consult with nurses about their health problems because the hierarchy between physicians and nurses is traditionally strong in the medical field. In this setting, the physician approves the treatment, and the nurse follows his or her instructions.^[51]^ Although a specific nurse-practitioner system has been created in Japan, it is thought that one of the reasons that few people consult with nurses is the difficulty in providing autonomous and self-reliant medical care by nurse practitioners, as in the US. Another factor might be the lack of a systematic CAM education program for nursing care. However, many CAM therapies, such as massages and touching-based therapies (which are performed to relieve patients’ physical and mental pain) include nursing care practices that many nurses have been already performing without being aware of their various benefits. These CAM practices relieve not only physical pain but also provide certain psychological and social benefits, thereby improving peoples’ QOL. For nurses who are interested in practicing CAM therapies to incorporate them into their daily care practices, the relevant knowledge and skills must be acquired, along with the availability of more opportunities to learn about CAM-based therapies.

This study found that a person’s choice of CAM therapy varied depending on the health problem being experienced. The I-CAM-Q questionnaire also identified the perceived purpose and usefulness of various CAM therapies according to our respondents. Often, our participants chose a CAM therapy that would relieve or treat the symptoms involved in either the temporal or the causal development of their health problems. Most of the specialists, whom the participants consulted with regarding their health problems, were physicians; therefore, it is pertinent for physicians to understand the CAM therapies that patients are using or requesting to use so that they can formulate an effective treatment plan. People also consult frequently with massage therapists about various health problems. Often, a massage can take longer for a single treatment compared to a typical physician’s visit. As there is an increased focus on building interpersonal relationships, people are more likely to prefer longer sessions. Additionally, nurses are known to play an important role as healthcare professionals who are in direct contact with patients, meaning that it is important to educate them about CAM therapies, making it easier for them to consult with patients around these kinds of treatment plans.

## 5. Limitations

This study has 5 limitations. First, we adopted a cross-sectional design, meaning that we could not examine any temporal or causal relationships between our variables. Second, it might be difficult to generalize our findings because our target population only consisted of people who were specifically interested in diet and health. Third, we assumed that participants who were explicitly interested in CAM therapies would be more likely to answer the questionnaire than would those who were not interested. It was also assumed that participants who were not familiar with computers or the Internet might have difficulty answering the study questions. As such, these factors may have caused a selection bias. Fourth, if participants did not understand the questions or had queries when answering the questionnaire, they were not able to discuss these with the researchers because it was not a face-to-face interview survey. As such, some may have answered inappropriately. These factors might also be related to the low response rates observed in our study (18.9%). Finally, this study’s sample size was relatively small because of the low response rate. Therefore, further research ensuring a high response rate must be conducted. Based on these limitations, future studies should adopt a longitudinal design, involve more participants, and adopt different methodologies, such as face-to-face interviews or focus groups.

## 6. Conclusion

This study used the I-CAM-Q, which is an internationally standardized questionnaire, to investigate Japanese participants’ use of CAM therapies for different health problems. The self-help practices related to the identified CAMs differed for specific health problems, such as musculoskeletal and heart diseases, with the perceived purpose and usefulness of the utilized CAM therapies being clarified with the respondents. Additionally, the most common professionals consulted for health problems were found to be physicians. However, when asked whether it was helpful to consult with a specific professional for health problems, the percentage of participants who answered “very helpful” was higher for massage, judo, acupuncture, and moxibustion therapists, than for physicians. The results of this study will help to inform medical providers of the most appropriate types of CAMs for dealing with various health problems.

## Acknowledgments

The authors thank Mr. Takeshi Hataoka, the secretariat of the JAMBC, for his cooperation in compiling the questionnaire. The authors also thank Ms. Yayoi Tagawa, the secretariat of the University of Tokushima Graduate School of Biomedical Science, for her valuable secretarial support. We would like to thank Editage (www.editage.com) for English language editing.

## Author contributions

**Conceptualization:** Ryo Tabata, Kenji Tani.

**Data curation:** Ryo Tabata, Harutaka Yamaguchi.

**Formal analysis:** Ryo Tabata, Harutaka Yamaguchi.

**Funding acquisition:** Ryo Tabata, Kenji Tani.

**Investigation:** Ryo Tabata.

**Methodology:** Ryo Tabata, Yoshihiro Ookura.

**Project administration:** Kenji Tani.

**Resources:** Harutaka Yamaguchi, Kenji Tani.

**Software:** Ryo Tabata, Harutaka Yamaguchi.

**Supervision:** Kenji Tani.

**Validation:** Yoshihiro Ookura.

**Visualization:** Ryo Tabata, Harutaka Yamaguchi, Yoshihiro Ookura, Kenji Tani.

**Writing – original draft:** Ryo Tabata, Harutaka Yamaguchi, Yoshihiro Ookura, Kenji Tani.

**Writing – review & editing:** Ryo Tabata, Harutaka Yamaguchi, Yoshihiro Ookura, Kenji Tani.
